# Editorial: Unlocking marine food potentials: empowering bioactive compounds, precision nutrition, and sustainable dietary solutions

**DOI:** 10.3389/fnut.2025.1674047

**Published:** 2025-08-18

**Authors:** Weiyang Zhao, Yifeng Zhang, Han Sun

**Affiliations:** ^1^School of Biological Sciences, University of Hong Kong, Hong Kong, Hong Kong SAR, China; ^2^Department of Food Safety and Health, School of Advanced Agricultural Sciences, Peking University, Beijing, China; ^3^Engineering Research Center of Watershed Carbon Neutrality of Ministry of Education, and Center for Algae Innovation & Engineering Research, School of Resources and Environment, Nanchang University, Nanchang, China

**Keywords:** marine food, microalgal, bioactive compounds, dietary solution, nutrition

Contemporary food systems are under increasing strain due to the rapid expansion of the global population, shifting dietary preferences, and growing awareness of the environmental impacts associated with traditional agricultural practices (1). While the land has long been the primary focus for food production, it is becoming increasingly clear that terrestrial resources alone may be insufficient to meet future nutritional needs in a manner that is both sustainable and health-promoting. In response to this challenge, attention has turned toward the marine environment as a vast and largely untapped reservoir of functional food components and high-value nutrients.

Marine ecosystems encompass an extraordinary diversity of life forms, many of which produce bioactive compounds with demonstrated physiological benefits. These compounds include, but are not limited to, long-chain omega-3 polyunsaturated fatty acids (such as EPA and DHA), bioactive peptides, sulfated polysaccharides, and a wide array of natural pigments and antioxidants. Their roles in modulating lipid metabolism, reducing inflammation, improving gut microbiota composition, and supporting immune function are being increasingly recognized in both experimental and clinical settings (2).

In parallel with this scientific interest, technological advancements have made it more feasible to isolate, purify, and formulate marine-derived compounds for functional food and nutraceutical applications (3). Environmentally conscious extraction methods such as subcritical fluid extraction, molecular distillation, and ultrasound-assisted processing are offering more sustainable alternatives to conventional techniques, aligning with global goals for reducing ecological footprints (4).

Furthermore, marine bioresources offer opportunities beyond human nutrition. Innovations in aquaculture, integrated waste management, and biostimulant development demonstrate how marine-derived materials can contribute to circular economy models and regenerative agricultural systems. These synergies between nutritional efficacy and sustainability potential form a compelling argument for expanding research and investment into marine-based food innovations.

The selected contributions presented in this Research Topic exemplify diverse strategies for leveraging marine resources in both health-focused and sustainability-driven contexts. Meng et al. developed and validated a novel extraction protocol for obtaining lipids rich in eicosapentaenoic acid (EPA) from *Nannochloropsis gaditana* using subcritical butane and molecular distillation. This environmentally conscious method yielded a highly purified extract with an EPA content of 58.92% (w/w), significantly higher than many conventional techniques. In a hyperlipidemic mouse model, supplementation with the purified lipid extract led to notable improvements in lipid profiles, including reductions in body weight gain, serum triglycerides, total cholesterol, and LDL-C, alongside an increase in HDL-C. Furthermore, the extract demonstrated anti-inflammatory effects by lowering hepatic levels of TNF-α and IL-1β, and reduced oxidative stress through enhanced activities of SOD, CAT, and GSH-Px. Key lipid metabolism enzymes were also favorably modulated. This study not only highlights the therapeutic potential of microalgal EPA but also exemplifies how advanced extraction technologies can enhance the functional quality of marine-derived compounds.

Jiang et al. investigated the digestion and fermentation behavior of sulfated polysaccharides from the brown seaweed *Sargassum fusiforme*. *In vitro* digestion studies indicated that the polysaccharides remained intact through the gastrointestinal tract, reaching the colon without releasing free monosaccharides. Fermentation by human gut microbiota led to selective utilization of higher molecular weight fractions, resulting in increased production of short-chain fatty acids (SCFAs), including acetate, propionate, and butyrate. SFSP treatment also altered the microbial community composition, promoting beneficial taxa such as *Bacteroides* and *Megamonas*, while suppressing potential pathogens like *Shigella* and *Klebsiella*. Metabolomic analysis revealed increased levels of bioactive organic acids and reduced concentrations of harmful compounds such as trimethylamine and secondary bile acids. These findings support the prebiotic potential of SFSP and highlight its ability to modulate gut ecology and host metabolism through microbiota-mediated pathways.

Dai et al. addressed the challenge of managing the residual supernatant generated from microalgae cultivation by exploring its application as a biostimulant in agriculture. The researchers evaluated the effects of supernatants from *Chlorella vulgaris* cultured under autotrophic, heterotrophic, and mixotrophic conditions on lettuce (*Lactuca sativa*) growth. While autotrophic cultures yielded supernatants richer in inorganic nutrients, those from heterotrophic and mixotrophic cultures contained higher levels of amino acids and plant hormones. Application of these supernatants significantly enhanced lettuce root and shoot biomass, chlorophyll concentration, soluble sugars, and protein content, while reducing nitrate accumulation. The study offers a compelling case for integrating algal cultivation by-products into plant production systems, fostering resource efficiency, and contributing to sustainable agriculture through nutrient recycling and waste minimization.

Cropotova et al. explored the post-processing enhancement of fish protein hydrolysates (FPH) derived from Atlantic mackerel (*Scomber scombrus*) by applying ultrasound treatments at different power levels (300W, 450W, and 600W). The ultrasound exposure significantly improved protein solubility and degree of hydrolysis, and led to an increase in antioxidant capacity as measured by DPPH, FRAP, and ABTS assays. Moreover, the FPH exhibited stronger inhibition of dipeptidyl peptidase-IV (DPP-IV), an enzyme involved in glucose metabolism. Cell-based assays using human Caco-2 cells further demonstrated the hydrolysates' ability to reduce oxidative stress and lipid peroxidation under H_2_O_2_-induced conditions. These results underscore the effectiveness of non-thermal ultrasound processing in enhancing the nutritional and functional quality of marine protein ingredients and reinforce the value of fishery by-products as sources of health-promoting compounds.

These contributions collectively illustrate the vast potential of marine-derived compounds in driving forward health and sustainability agendas. Their findings reflect a convergence of technological innovation, nutritional science, and ecological consciousness. The refinement of extraction and processing methods, such as subcritical fluid techniques and ultrasound-assisted treatment, enables not only higher yields and purity but also improved bioactivity profiles, making marine resources more competitive with traditional sources.

In addition, the biological effects observed—from lipid modulation and antioxidative activity to microbiota composition shifts and plant biostimulation—highlight the multifunctionality of marine bioactives. These compounds do not serve a single purpose but operate across multiple physiological domains, opening avenues for integrated applications in food, healthcare, and agriculture. Importantly, by transforming waste streams into valuable inputs, several studies demonstrate circular economy principles in action, suggesting scalable models for sustainable production.

As the search for climate-resilient, nutritionally optimized food systems intensifies, the ocean's role as a provider of diverse, renewable, and health-enhancing compounds is becoming increasingly evident. Further research, collaboration, and policy alignment will be essential to fully realize the promise of the marine biosphere in global nutrition and sustainability strategies.

While the research discussed in this editorial highlights significant progress in the discovery, extraction, and application of marine bioactive compounds, several avenues remain for further development. One of the critical next steps involves the translation of laboratory and preclinical findings into human clinical trials. Establishing efficacy, safety, and dosage in real-world settings is essential for integrating these compounds into mainstream health and nutrition protocols.

Mechanistic studies are also warranted to elucidate how these bioactives interact with host systems at the molecular level. Understanding pathways of action—whether through modulation of gene expression, receptor binding, enzymatic activity, or microbial interactions—will support targeted nutritional interventions and personalized dietary solutions. In addition, standardization of extraction techniques and bioactivity assays will help ensure consistency and reproducibility across studies. Regulatory frameworks will need to evolve in tandem, supporting innovation while safeguarding public health.

There is also a compelling need to integrate marine bioresources into broader food system planning. Strategies that link marine-based innovations with terrestrial agriculture, renewable energy, and environmental stewardship can foster more resilient and circular production models (5). Interdisciplinary collaboration—combining expertise in marine biology, food science, health, economics, and policy—will be indispensable in unlocking the full potential of the ocean for sustainable nutrition. By continuing to explore and responsibly harness the ocean's rich biodiversity, we can pave the way toward more sustainable, health-oriented, and inclusive food systems that benefit both people and the planet.
